# Confusion will be my epitaph: genome-scale discordance stifles phylogenetic resolution of Holothuroidea

**DOI:** 10.1098/rspb.2023.0988

**Published:** 2023-07-12

**Authors:** Nicolás Mongiardino Koch, Ekin Tilic, Allison K. Miller, Josefin Stiller, Greg W. Rouse

**Affiliations:** ^1^ Scripps Institution of Oceanography, University of California San Diego, La Jolla, CA, USA; ^2^ Department of Marine Zoology, Senckenberg Research Institute and Museum, Frankfurt, Germany; ^3^ Anatomy Department, University of Otago, Dunedin, Otago, New Zealand; ^4^ Centre for Biodiversity Genomics, Section for Ecology and Evolution, Department of Biology, University of Copenhagen, Copenhagen, Denmark

**Keywords:** sea cucumbers, systematics, phylogenomics, phylogenetic signal, phylogenetic conflict

## Abstract

Sea cucumbers (Holothuroidea) are a diverse clade of echinoderms found from intertidal waters to the bottom of the deepest oceanic trenches. Their reduced skeletons and limited number of phylogenetically informative traits have long obfuscated morphological classifications. Sanger-sequenced molecular datasets have also failed to constrain the position of major lineages. Noteworthy, topological uncertainty has hindered a resolution for Neoholothuriida, a highly diverse clade of Permo-Triassic age. We perform the first phylogenomic analysis of Holothuroidea, combining existing datasets with 13 novel transcriptomes. Using a highly curated dataset of 1100 orthologues, our efforts recapitulate previous results, struggling to resolve interrelationships among neoholothuriid clades. Three approaches to phylogenetic reconstruction (concatenation under both site-homogeneous and site-heterogeneous models, and coalescent-aware inference) result in alternative resolutions, all of which are recovered with strong support and across a range of datasets filtered for phylogenetic usefulness. We explore this intriguing result using gene-wise log-likelihood scores and attempt to correlate these with a large set of gene properties. While presenting novel ways of exploring and visualizing support for alternative trees, we are unable to discover significant predictors of topological preference, and our efforts fail to favour one topology. Neoholothuriid genomes seem to retain an amalgam of signals derived from multiple phylogenetic histories.

## 1. Introduction

**Figure 1.**
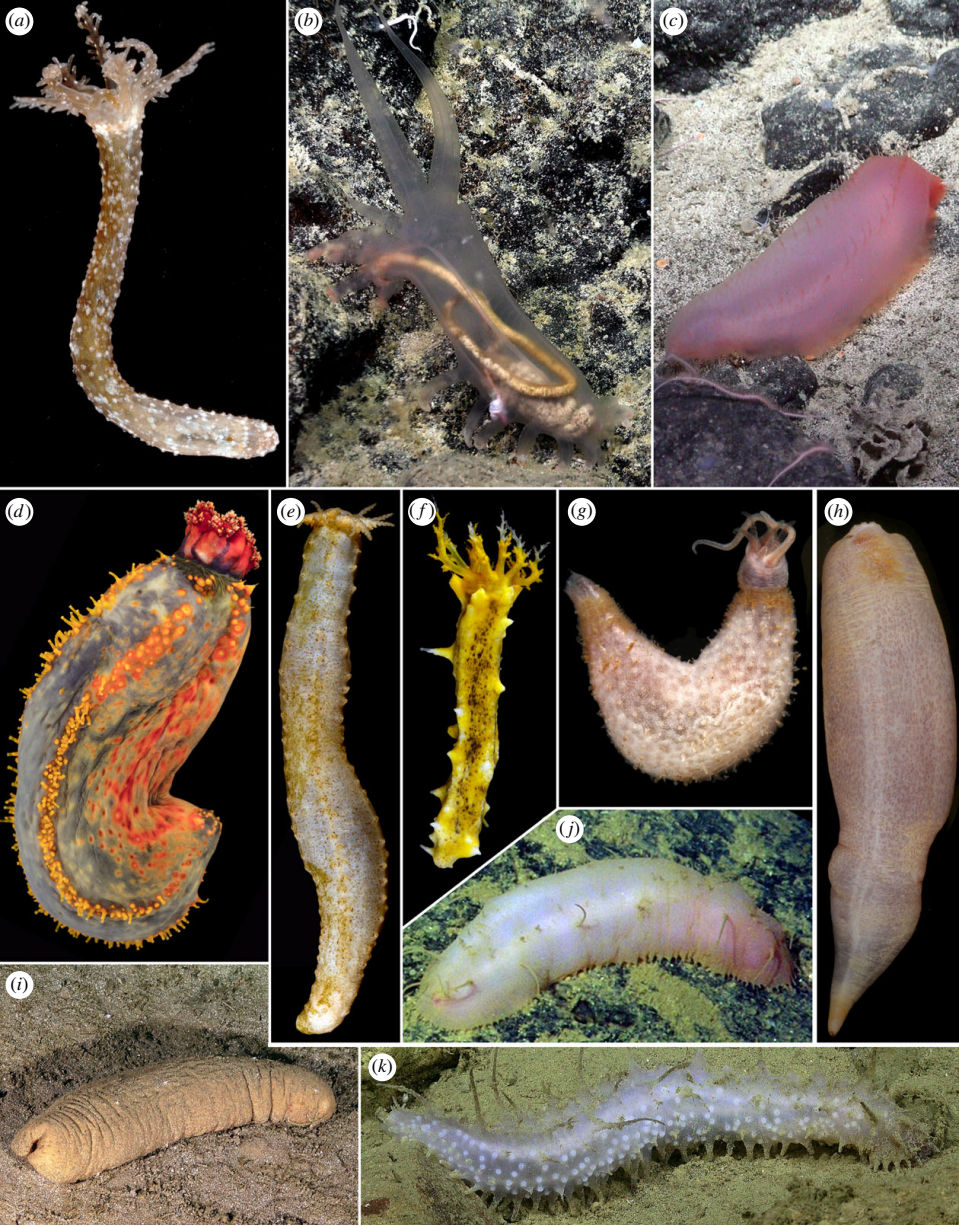
Representative holothuroid diversity included in this study. (*a*). *Synapta* sp. (*b*). *Peniagone* cf. *vitrea*. (*c*). *Benthogone* sp. (*d*). *Pseudocolochirus violaceus*. (*e*). *Abyssocucumis albatrossi*. (*f*). *Colochirus robustus*. (*g*). *Ypsilothuria* n. sp. (SIO-BIC E6221). (*h*). *Molpadia amorpha*. (*i*). *Pseudostichopus* cf. *mollis*. (*j*). Synallactidae. (*k*). *Bathyplotes* cf. *moseleyi*. The classification of these terminals can be found in electronic supplementary material, table S1. All photos except (*g*) are of the voucher specimens sequenced (catalogue numbers can be found in electronic supplementary material, table S1; further sampling information is accessible through the SIO-BIC online database, https://sioapps.ucsd.edu/collections/bi/). Images (*b*), (*c*), (*i*) and (*k*) are courtesy of the Schmidt Ocean Institute, and image (*j*) is courtesy of Monterey Bay Aquarium and Research Institute.

Holothuroidea (commonly known as sea cucumbers) is arguably the most morphologically diverse major clade of extant Echinodermata (figure 1). The smallest adults can be less than 1 cm in length, as seen in the meiofaunal *Leptosynapta minuta* [1] and epibenthic *Incubocnus* [2]. The largest can be thin and elongate, reaching several metres length, as in the snake sea cucumber *Synapta maculata* [3], or they may be less than a metre but robust and weighing over 5 kg, as in the case of *Holothuria fuscopunctata* [4]. While predominantly benthic as adults, some taxa are capable of swimming and there are even forms that spend their entire lives in the water column, as *Pelagothuria natatrix* does [5]. While all holothuroids have a ring of tentacles and are deposit or filter feeders, some clades lack tube feet and have a substantially reduced water vascular system, traits otherwise developed across all echinoderms. They can also entirely lack calcareous elements (ossicles) in the body wall, or these can be expanded to form overlapping plates that build a rigid test [6]. There are currently 1775 accepted extant species of holothuroids [7] found in ocean waters that range from the intertidal to the bottom of the deepest trenches [8,9]. Especially in benthic deep-sea habitats, they can constitute the vast majority of total biomass and have a strong impact on ecosystem functioning, bioturbation and nutrient cycling [10–12].

While multiple morphological attempts have been made to delineate major subdivisions within Holothuroidea, these have been limited by the extreme simplification of their skeleton (relative to other echinoderms), the delicate and fragile nature of their bodies (which often results in poorly preserved specimens for morphological analyses) and the small number of traits that provide useful information at high taxonomic levels [13–15]. The most recent revision of the group's classification was based on a six-gene dataset including terminals from 25 of the 29 accepted family ranked taxa [16]. This study recovered a basal split within sea cucumbers between Apodida, a clade characterized by a complete loss of tube feet, and Actinopoda (among which secondary reductions or loss of tube feet occur only within Molpadida). Actinopoda were further subdivided into Elasipodida and Pneumonophora, the latter of which includes all species with respiratory trees, a unique cloacal invagination that plays an important (although not exclusive) role in respiration [17,18]. Furthermore, the names Holothuriida and Neoholothuriida were applied to the main subdivisions within Pneumonophora, with four well-supported major clades inside Neoholothuriida: Dendrochirotida, Molpadida, Persiculida and Synallactida. However, the relationships among these four lineages remained uncertain. A phylogenetic resolution for the major neoholothuriid lineages is necessary to explore the origins of the high morphological and ecological disparity harboured by this clade, as well as to establish a natural classification framework for a substantial fraction of sea cucumber diversity (62% of species-level diversity is contained within Neoholothuriida [7]). Miller *et al*. [16] concluded that meeting these objectives would likely require sequencing efforts of a different magnitude.

Here we present the first phylogenomic study of sea cucumbers, the last major eleutherozoan clade (which further includes echinoids [19], asteroids [20] and ophiuroids [21]) to have its phylogeny tackled using genome-scale datasets. Through the generation of novel transcriptomic resources for holothuroids we built a molecular dataset encompassing over a thousand orthologues. The goal was to resolve some of the lingering uncertainties in the holothuroid tree of life, yet a continuing lack of resolution encouraged novel ways to explore phylogenomic datasets.

## 2. Material and methods

### (a) Taxon sampling, extraction and sequencing

Sea cucumber specimens were collected by SCUBA diving, snorkeling, dredging and remotely operated vehicles (ROV), or purchased from aquarium suppliers. Specimen collection and fieldwork was performed under permits whenever applicable. All vouchers were deposited at the Benthic Invertebrate Collection, Scripps Institution of Oceanography (SIO-BIC; electronic supplementary material, table S1). Species identification was based on multiple lines of evidence, including anatomical (gross and ossicle morphology), biogeographical and molecular (mitochondrial cytochrome c oxidase subunit I, COI) information. DNA extractions and COI amplifications followed protocols described in Miller *et al*. [16], and sequences are deposited in NCBI (accession numbers available in electronic supplementary material, table S1). In the case of unavailable COI sequences, these were mined from assembled transcriptomes by blasting against close relatives. Previous identifications of transcriptomic vouchers at SIO were also revised, including those sequenced and released as part of EchinoDB [22,23].

For large specimens, tissue was dissected from the body wall or tube feet, while whole body sections were sampled for the remaining samples. Sampled tissues were finely chopped, placed in RNA*later* (Invitrogen) buffer solution, and stored at −80°C. RNA extractions were performed from Trizol (Thermofisher), using Direct-zol RNA Miniprep Kit with in-column DNAse treatment (Zymo Research). mRNA was isolated with Dynabeads mRNA Direct Micro Kit (Invitrogen). mRNA concentration was estimated using Qubit RNA broad range assay kit (Thermofisher), and quality was assessed using RNA ScreenTape with an Agilent 4200 TapeStation on an Agilent Bioanalyzer 2100. Most libraries were prepared using a KAPA-Stranded RNA-Seq kit targeting a 200–300 bp insert size, and results were assessed using DNA ScreenTape (Bioanalyzer 2100). Libraries were then sequenced in multiplexed (8 libraries per lane) pair-end runs using 150 bp paired-end Illumina HiSeq 4000 at the UC San Diego IGM Genomics Center. To minimize read crossover, we employed 10 bp sequence tags designed to be robust to indel and substitution errors [24]. For four samples (*Benthodytes* cf. *sanguinolenta*, *Benthogone* sp., *Colochirus robustus* and *Peniagone* cf. *vitrea*), library preparation and multiplexed pair-end sequencing on an Illumina NovaSeq 6000 PE150 was performed by Novogene.

Thirteen novel transcriptomes were generated for this study and combined with publicly available genomic and transcriptomic datasets downloaded from NCBI and EchinoBase [25]. Final taxonomic sampling included 35 holothuroids as well as three echinoid and one asteroid outgroups (electronic supplementary material, table S1). Raw files for all novel datasets, as well as those so far available only on EchinoDB [22], are deposited in the NCBI sequence read archive (SRA) under BioProject PRJNA979278. All assemblies are available at the associated Dryad data repository (doi:10.5061/dryad.0p2ngf255) [26], along with the phylogenetic matrices, trees and other results derived from them.

### (b) Assembly, sanitation and matrix construction

Reads were trimmed or excluded based on quality scores using Trimmomatic v 0.3.6 under default settings [27]. Additional sanitation steps were implemented by the Agalma 2.0 pipeline [28], resulting in the removal of reads based on compositional and quality filtering criteria, as well as those mapping to rRNA sequences or retaining adapter sequences. Remaining reads were assembled *de novo* with Trinity v. 2.5.1 [29]. Assemblies were then screened for contaminants using alien_index v 3.0 [30]. Transcripts with substantially better BLAST + [31] hits to a dataset of well-curated archaeal, bacterial and fungal genomes than to a metazoan database (both available from http://ryanlab.whitney.ufl.edu/downloads/alien_index/), defined as those exhibiting an alien index >45 (see [32]), were excluded. Sanitized transcriptomes were imported back into Agalma for orthology inference [28,33], which included tree-based steps to refine orthologues by identifying duplication events on gene trees and pruning putative paralogous sequences. Alignment and quality-based trimming were performed with MAFFT v. 7.305 [34] and GBLOCKS v. 0.91b [35]. The resulting supermatrix was reduced using a 70% occupancy threshold, resulting in a dataset composed of 1159 orthologues coded as amino acids (from a total of 13 767).

Gene trees were inferred from each amino acid alignment with ParGenes v. 1.0.1 [36], using optimal models and 100 bootstrap replicates. These were analysed with TreeShrink v. 1.3.1 [37] (using parameters −q 0.01 −k 3 −b 25), which employs taxon-specific distributions of root-to-tip distances to identify outlier sequences potentially suffering from errors in alignment or orthology inference. Identified outliers were removed from both gene trees and individual alignments, and a new supermatrix was concatenated. As a final sanitation step, the data was run using *genesortR* [38,39], which ordered all loci based on decreasing estimates of phylogenetic usefulness (electronic supplementary material, figure S1). The worst-ranked 59 loci (5.1% of supermatrix) were further discarded, resulting in a final dataset of 1100 loci and 264 991 amino acid positions. Two smaller datasets, composed of the top-scoring (i.e. most phylogenetically useful) half and quarter of loci (550 and 225, respectively) were also output for analysis.

### (c) Phylogenetic inference

Phylogenies were inferred from all three datasets using a variety of approaches. First, gene trees were provided to the coalescent-aware summary method ASTRAL-III [40], which employs local posterior probabilities to estimate node support [41]. Second, tree inference was performed under a best-fit partitioned model with IQ-TREE 2 v. 2.1.3 [42–44], using the fast-relaxed clustering algorithm to merge individual loci (using parameters -m MFP + MERGE -rclusterf 10 -rcluster-max 3000). Finally, the site-heterogeneous model CAT-PMSF [45] was used as an efficient alternative to the computationally demanding CAT model family [46]. For each dataset, short runs of 1100 generations were done in PhyloBayes-MPI v. 1.8.1 [47] under a fixed topology (that obtained with ASTRAL-III) to approximate site-specific stationary distributions and amino acid exchangeabilities under the CAT + GTR model. Model parameters were summarized after discarding the initial 100 generations as burn-in, and reformatted using scripts available at https://github.com/drenal/cat-pmsf-paper. Tree inference was then performed in IQ-TREE 2 under maximum likelihood using the PMSF method [48], setting exchangeabilities and site-specific frequencies to the posterior mean estimates previously obtained with PhyloBayes. For both concatenation approaches, support was estimated using 1000 replicates of ultrafast bootstrap [49].

### (d) Phylogenetic signal dissection

Several avenues were explored to assess levels of phylogenetic signal and conflict in the data. First, a treespace was built from gene trees using quartet dissimilarities to estimate topological differences and principal coordinate analysis (PCoA) as the method of ordination [50]. Levels of incongruence (henceforth, topological disparity) were compared between the complete and usefulness-based subsampled datasets using averaged Euclidean distances to the centroid. Values for subsampled datasets were then compared against null distributions built using 1000 replicates of randomly selected one half and one quarter of gene trees. This approach relied on R packages *Quartet* [51] and *dispRity* [52]. Loci were also characterized by their ability to recover several deep, well-supported and non-nested nodes: Apodida, Elasipodida, Holothuriida, Synallactida and Dendrochirotida (i.e. all currently recognized order-level clades [7] represented by at least three sampled terminals). Gene trees recovering a large proportion of ‘uncontroversial’ nodes have been considered less likely to suffer from hidden paralogy and more prone to retain true phylogenetic signals [53,54]. For each loci, results were summarized using the proportion of clades recovered from among those whose monophyly could be tested (i.e. those represented by at least two terminals).

Since there was persistent discordance among methods of inference regarding the resolution of major neoholothuriid clades, phylogenetic signal for the alternative topologies was explored using site-wise log-likelihood scores. Scores were computed with IQ-TREE 2 under the best-fit partitioned model and using three constrained topologies differing only in the position of Molpadida (the remaining incongruence was fixed to the preferred resolution, see Results). Gene-wise log-likelihood scores were obtained by adding scores across sites, and their differences for all pairs of topologies—known as *Δ*GLS values—were computed. Given linear dependency between the three ΔGLS values (Δ*GLS_x_* = Δ*GLS_y_* − Δ*GLS_z_*), these were visualized in a two-dimensional space which was rotated using principal components analysis (PCA). A nominal ΔGLS threshold of ±2 log-likelihood units was used to categorize loci as either informative or uninformative with regards to a given topological comparison. The relative enrichment for/against alternative neoholothuriid topologies was assessed in subsampled matrices enriched in both phylogenetic usefulness and loci recovering high proportions of ‘uncontroversial’ nodes (see above).

To explore the drivers of differences in ΔGLS across loci, fifteen gene properties were estimated and treated as potential determinants. These were all calculated by *genesortR* [39], and included commonly used metrics of phylogenetic signal, potential sources of systematic bias, and estimates of the overall information content and evolutionary rate of each individual loci. Further details on these metrics can be found in electronic supplementary material, table S2.

Potential links between gene properties and the phylogenetic support for alternative neoholothuriid relationships were explored using two different statistical approaches. Associations between the PCA axes derived from ΔGLS values (representing major aspects of phylogenetic signal for competing hypotheses) and explanatory variables were first tested using the ‘envfit’ function in R package *vegan* [55]. This approach overlayed vectors onto the ordination plot depicting the directions and magnitudes of maximum correlation between individual gene properties and PCA scores. Each predictor was analysed separately, and the significance of the correlations tested using 10 000 random permutations. Given the possibility of nonlinear relationships between predictor and response variables, a second approach was explored in which *Δ*GLS values were transformed into a single categorical factor with three levels. For this, loci were categorized into: (A) uninformative, including those for which all ΔGLS were within ±2 log-likelihood units; as well as those either (B) supporting or (C) rejecting the resolution obtained using ASTRAL-III, defined as exhibiting at least one comparison favouring or disfavouring such topology, respectively, by an absolute ΔGLS value >2. This categorization is supported by analyses showing that the main aspect of differences in phylogenetic signal across loci relates to their support for/against the topology obtained with ASTRAL-III, with very little ability to discriminate between the two other alternatives (see Results). A conditional inference classification tree was fit to the data using function ‘ctree’ in R package *partykit* [56], assessing whether partitioning the data by values of any of the gene properties was able to generate subsets of loci that show similar topological preferences. A Bonferroni correction for multiple comparisons was applied, and significant predictors were visualized on the ordination plot using smooth surfaces fit using penalized regression splines [57].

All statistical analyses were performed in the R environment v. 4.2.2 [58] using code reliant on the packages mentioned above, as well as *adephylo* [59], *ape* [60], *phangorn* [61], *phytools* [62] and those included in the *tidyverse* [63].

## 3. Results

**Figure 2.**
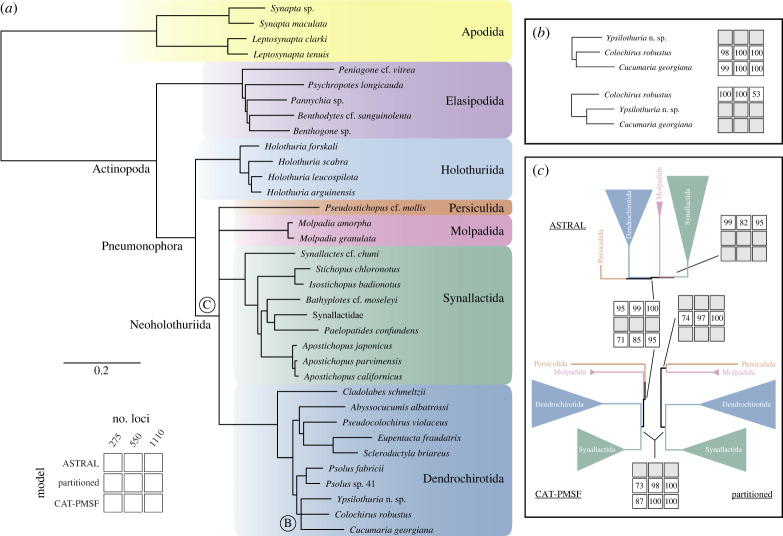
Summary of phylogenetic inference results. (*a*). Strict consensus of the nine inference conditions explored, varying both the number of loci and the method of inference. Nodes disagreeing between analyses are collapsed and labelled (see panels (*b,c*) for further details); branch lengths are otherwise taken from the CAT-PMSF analysis of the full dataset. (*b*). Monophyly of a clade composed of two cucumariid terminals, *Colochirus robustus* and *Cucumaria georgiana*, is rejected by ASTRAL-III, but upheld by the other methods (legend for support value grid is shown in (*a*)). (*c*). Systematic disagreement between all methods of inference regarding relationships among major neoholothuriid clades. The resolution favoured by each method is found across datasets of different sizes. Topologies, branch lengths and support values for each individual analysis are shown in electronic supplementary material, figures S2–S4.

Phylogenetic inference under all methods explored and for the three datasets of different sizes recovered highly congruent and well-supported topologies (electronic supplementary material, figures S2–S4), which were also in broad agreement with the most recent large-scale study based on Sanger-sequenced loci [16]. As summarized in figure 2*a*, Apodida, Elasipodida and Holothuriida formed successive and monophyletic sister groups to the remainder of sea cucumber diversity included within Neoholothuriida. The latter was further subdivided into four major lineages: Dendrochirotida, Molpadida, Persiculida and Synallactida. Nodes defining all aforementioned clades had maximum support across analyses. Support for currently recognized order level clades was surprisingly unambiguous: 69.2% of gene trees resolved a monophyletic Synallactida, and between 87.1 and 96.9% recovered the monophyly of Apodida, Elasipodida, Holothuriida and Dendrochirotida (electronic supplementary material, figure S5). Despite the relatively small size of loci (mean number of characters: 239.9, range = 103–621), 88.0% simultaneously resolved at least two thirds of these clades, and 58.1% resolved them all.

Only two regions of the tree topology showed incongruent resolutions among the analyses performed (figure 2*b,c*). First, ASTRAL-III rejected a close relationship among two of the cucumariid species sampled, *Colochirus robustus* and *Cucumaria georgiana*, which otherwise formed a clade under concatenation approaches (figure 2*b*). Given the otherwise unambiguous support for a close relationship between *Colochirus* and *Cucumaria*, as well as the poor node support for the ASTRAL-III topology when using the complete dataset, we tentatively favour here the results obtained under concatenation methods. We note, however, that a monophyletic Cucumariidae was not recovered by our analyses regardless of how these terminals are resolved, as they were only distantly related to the remaining cucumariids (*Abyssocucumis*, *Pseudocolochirus*). In fact, discrepancies between our trees and the current family level classification of sea cucumbers are pervasive, and also included the non-monophyly of elasipodid families Psychropotidae (*Psychropotes*, *Benthodytes*) and Laetmogonidae (*Benthogone*, *Pannychia*), the dendrochirotid family Sclerodactylidae (*Cladolabes*, *Eupentacta*, *Sclerodactyla*), and the synallactid families Synallactidae (*Synallactes*, *Bathyplotes*, *Paelopatides*) and Stichopodidae (*Stichopus*, *Isostichopus*, *Apostichopus*).

A second and more striking topological discordance involved the organization of the four major lineages within Neoholothuriida (figure 2*c*). Each one of the different methods of inference proposed an alternative resolution for the clade, which were recovered regardless of dataset size and strongly supported (values >95) when employing the complete supermatrix. While all three inference methods agreed on a subtree in which Dendrochirotida and Synallactida share a closer relationship than either one does with Persiculida, the position of Molpadida within this scaffold was highly unstable and methodologically sensitive. Supported alternatives included a placement of Molpadida as sister to either Synallactida, Persiculida, or Synallactida + Dendrochirotida (henceforth referred to as ‘ASTRAL’, ‘partitioned’, and ‘CAT-PMSF’ topologies, respectively; figure 2*c*). Despite this level of uncertainty, our analyses still reject the long-hypothesized close relationships between Molpadida and Dendrochirotida [13,15], as well as the topology of Miller *et al*. [16] in which Dendrochirotida placed as sister group to all other neoholothuriids. While support values for some deep nodes decreased when performing inference with the smallest of datasets, this seems to be entirely driven by a reduction in the amount of data, as subsampled matrices showed significant reductions in overall phylogenetic conflict (estimated using topological disparity; electronic supplementary material, figure S6).

**Figure 3.**
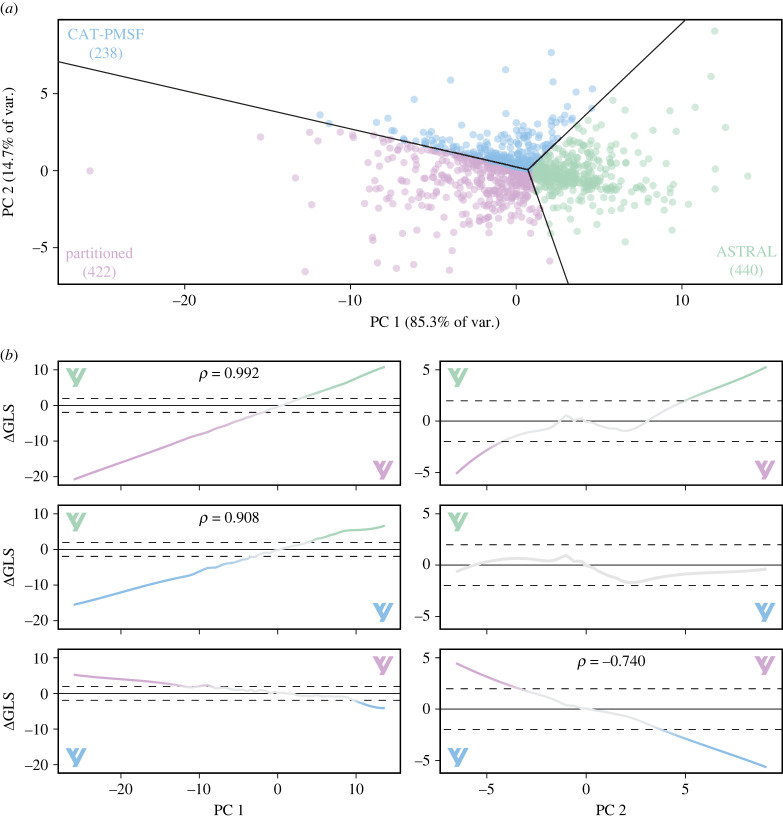
Exploration of support for alternative neoholothuriid topologies across loci. (*a*). Principal components (PC) axes obtained from the three ΔGLS. Percentages of explained variance are shown on axis labels. Loci are colour coded depending on their favoured topology. (*b*). Relationship between the PC axes and the scores of individual ΔGLS. Trendlines correspond to LOESS smoothing curves, and *ρ*-values (Spearman's rank correlation coefficients) are shown when absolute values > 0.7, taken to represent strong correlations. The area included within ± 2 log-likelihood units is highlighted and considered an area of weak support. Note the markedly different scales of the *y*-axes for PCs 1 and 2. Topologies are colour coded as in (*a*).

**Figure 4.**
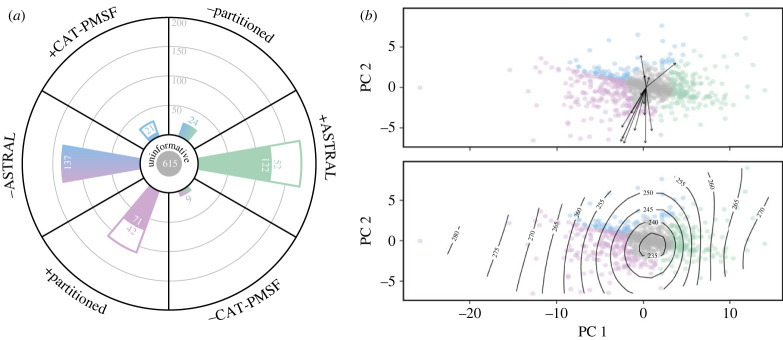
Categorization of loci depending on their favoured topology, and exploration of potential determinants. Colouring scheme follows that of figure 3. (*a*). Most loci (615, 55.9% of the full dataset) can be considered uninformative regarding relationships among neoholothuriid clades. The remainder can be classified into those supporting a given topology (denoted using a plus sign, +) if they favour a given resolution against both alternatives (coloured section of bar chart) or only one (white section of bar chart) with a ΔGLS ≥ 2; or rejecting a given topology (denoted using a minus sign, −). The number of loci either supporting (right side of wheel) or rejecting (left side of wheel) the ASTRAL topology are comparable in number: 207 (18.8%) versus 278 (25.3%), respectively. Further details on loci categorization can be found in electronic supplementary material, figure S7. (*b*). Top: Exploration of 15 potential determinants of ΔGLS. Arrows indicate directions of maximum correlation between scores and determinants; their length is scaled to the strength of the correlation. Predictors mostly load onto PC 2. R^2^ and *p*-values are shown in electronic supplementary material, table S2, but no correlation is significant. Bottom: Smoothed surface of alignment length, the only significant determinant found using a classification tree. Longer loci are more likely to be informative, yet alignment length does not predict which topology is preferred (see electronic supplementary material, figure S9).

To further explore the phylogenetic signal for competing neoholothuriid topologies, we estimated gene-wise log-likelihood scores for the three alternative resolutions of this clade. A PCA of differences in the scores obtained for pairs of topologies (ΔGLS) revealed that the topological preferences of loci could be summarized using a single major underlying axis which accounted for 85.3% of total variance (figure 3*a*). The scores of loci along this first PC axis represented the relative support either for or against the ASTRAL topology (figure 3*b*). On the other hand, the ability of loci to discern between the partitioned and CAT-PMSF trees was much weaker and mainly captured by the second PC axis, which explained only 14.7% of variance. The absolute values of ΔGLS were generally small, with most loci (615 loci, 55.9% of the complete dataset) being relatively uninformative regarding relationships among neoholothuriid clades (figure 4*a* and electronic supplementary material, figure S7). Nonetheless, the remainder of the dataset was once again roughly evenly split into a fraction that supported the ASTRAL configuration (207 loci, 18.8%), and one that rejected it in favour of either one, or both, of the topological alternatives (278 loci, 25.3%). These proportions remained stable across datasets subsampled using different strategies (electronic supplementary material, figure S8).

None of the 15 gene properties explored was recovered as a significant predictor of ΔGLS (figure 4*b*). Furthermore, these metrics correlated mostly with PC 2 (electronic supplementary material, table S2), leaving the major aspect of topological preference entirely unexplained. An alternative approach based on classification trees recovered one significant predictor: uninformative loci were significantly more likely to have a short alignment length, but this property also fails to explain which resolution was preferred by longer and more informative loci (electronic supplementary material, figure S9).

## 4. Discussion

Phylogenetic incongruence is a hallmark of genome-scale datasets [64–66]. A wide range of biological processes and methodological artefacts can lead phylogenomic datasets to harbour a mixture of phylogenetic signals, which can be differentially amplified by methods of reconstruction to produce conflicting, yet well-supported, topologies [67–69]. Different avenues have been proposed to ameliorate phylogenetic incongruence and favour a specific resolution for recalcitrant nodes. One strategy is to focus on data filtering, exploring the effects of removing sites and/or loci with unexpectedly high topological preferences [70,71], or those showing evidence of contributing mostly phylogenetic noise or biases [39,72]. Alternatively, methods have been developed to dissect alternative signals [73–75] in the hopes that one emerges as a better-justified option. Finally, exploring a range of inference methods, which vary in their realism, complexity, susceptibility to errors, and (potentially) relative fit, can also be used to justify favouring one among several alternative hypotheses [76–78]. However, even after exhaustive testing of these options, a robust resolution for some nodes on the tree of life remains elusive [79–82], awaiting the discovery of novel phylogenetic markers, improved taxon sampling or methodological developments.

We propose here that the early diversification of neoholothuriid sea cucumbers, an ancient, diverse and morphologically heterogeneous clade, constitutes another example of a group that defies phylogenetic resolution. Previous studies had acknowledged that a robust topology for Neoholothuriida was probably unattainable with the use of small molecular datasets [16], yet phylogenetic resolution remains out of reach even when employing more than a thousand loci. This result is particularly noteworthy given how comparatively trivial it is to correctly reconstruct other deep nodes (electronic supplementary material, figure S5). The reason underlying this uncertainty is not a lack of statistical power, but the presence of multiple signals supporting alternative trees. While a node uniting Dendrochirotida and Synallactida to the exclusion of Persiculida emerges from all our analyses, the position of Molpadida within this topology remains uncertain. A coalescent-aware method of reconstruction places Molpadida inside the clade containing Dendrochirotida and Synallactida, while concatenation-based methods place it outside, with further disagreement emerging depending on whether site-homogeneous or site-heterogeneous models are used. All three of these topological alternatives are well-supported and robust to gene subsampling, and thus represent an example of remarkable methodological sensitivity. Further exploration reveals that our dataset is unlikely to contain enough information to disambiguate between the topologies supported by alternative concatenation methods. On the other hand, the placements of Molpadida either inside or outside of the node containing Dendrochirotida + Synallactida are each strongly supported by substantial fractions of the data (19% and 25% of loci, respectively).

Complex site-heterogeneous models, such as the CAT family, are likely to fit genome-scale datasets better [48,83,84], but the use of model fit statistics when comparing mixture models against other alternatives (such as partitioned models) has been criticized [85]. Furthermore, issues relating to convergence, missing data, and over-parameterization [86–88] have still led many to question the results obtained under CAT models. Similarly, coalescent-aware methods have outperformed concatenation in a number of simulation scenarios [89,90], yet doubts remain regarding their usefulness to resolve ancient divergences, given that gene tree error is expected to surpass incomplete lineage sorting as the dominant source of incongruence for deep nodes [91]. The fit of summary methods (such as ASTRAL) is also impossible to evaluate relative to that of others, further complicating arriving at an objective way of preferring one method of inference from among those tested here.

In the absence of clear guidance as to which inference method should be preferred, we focused instead on evaluating the amount and quality of the signals supporting alternative placements of Molpadida. We used ΔGLS as proxies for the topological preference of loci (as in e.g. [19,70,92]), extending this type of analysis to simultaneously consider three alternative topologies. This allowed us to uncover a strong asymmetry in the ability of loci to distinguish between alternative trees and, as explained above, redirect our efforts to assessing two broad topological alternatives. Although many studies have succeeded in disentangling phylogenetic from non-phylogenetic signals by exploring loci quality [93–95], our attempts failed to find any determinants of topological preference: loci supporting alternative positions of Molpadida do not differ in their levels of phylogenetic signal, systematic biases, amounts of information or evolutionary rates. The only major pattern uncovered is that longer loci are more likely to harbour some sort of signal, a predictable and relatively trivial result stemming from the increased statistical power of longer alignments.

The branches subtending the major neoholothuriid clades are remarkably short (electronic supplementary material, figures S2–S4). This scenario, coupled with the deep origin of the major clades of crown holothuroids [16,96], is expected to result in unfavourable signal to noise ratios [97]. Nonetheless, the finding that substantial fractions of our supermatrix exhibit strong signal for competing topologies is not in line with Neoholothuriida originating from a hard polytomy [98]. Two broad explanations are therefore consistent with our results. First, incongruence might be caused by types of model violations that were not explicitly tested here, as would result, for example, from convergent shifts in amino acid composition [98–100]. Alternatively, neoholothuriid evolution might be better explained by ancient events of reticulation, as produced by processes such as ancient hybridization and incomplete lineage sorting. Finding direct evidence to substantiate these claims is complicated by the relatively sparse sampling attained by this study, the limited genomic resources available for the clade, and the lack of available methods that can simultaneously address both of these processes [79]. We suggest that Neoholothuriida constitutes a case of an ancient and rapid radiation, and further progress in its resolution could benefit from targeting data whose evolutionary history proves easier to model. Addressing the currently sparse sampling of both Molpadida and Persiculida should also be prioritized if we are to resolve this lingering uncertainty, especially through the sequencing of morphologically unique and potentially deeply divergent lineages such as Caudinidae, Eupyrigidae and Gephyrothuriidae.

Although the exact placement of Molpadida remains challenging to ascertain, phylogenomics reveals an otherwise robust higher-level topology for sea cucumbers. These efforts are a major step towards a stable classification for the group, corroborating much of the most recent classification based on a small-scale molecular dataset [16]. The results presented here constitute a necessary tool with which to elucidate the times of origin, morphological evolution and diversification dynamics of a major lineage of marine invertebrates. At the same time, they also show the extent to which the current family level classification scheme of holothuroids is at odds with their evolutionary history, highlighting the need for phylogenomic investigations with much-expanded taxon sampling and consequent morphological reassessments.

## Data Availability

COI sequences are available from GenBank under accession numbers OR082743-OR082756 and OR145350-OR145353; transcriptomic raw reads are available from SRA under BioProject PRJNA979278. All assemblies, phylogenomic datasets and trees, and other results, can be obtained from the Dryad Digital Repository: https://doi.org/10.5061/dryad.0p2ngf255 [26]. Supplementary figures and tables are provided in the electronic supplementary material [101].
